# The Changing Complementary Role of Multimodality Imaging in Clinical Cardiology

**DOI:** 10.3390/jcm11237095

**Published:** 2022-11-30

**Authors:** Gian Luigi Nicolosi

**Affiliations:** Cardiology, Policlinico San Giorgio, Via Gemelli 10, 33170 Pordenone, Italy; gianluigi.nicolosi@gmail.com

## 1. Introduction

Over the past two decades, major technological developments and progress have been reached for all imaging modalities applied to clinical cardiology, from echocardiography to magnetic resonance, computed tomography, nuclear imaging, etc. All these advancements and achievements progressively aid in filling the numerous gaps present in the current knowledge in the clinical practice of cardiology, as well as those of the disappointing unmet needs and goals for helping each patient, starting with clinical history, physical examinations and the standard 12-lead electrocardiogram (ECG) as a first step.

In this context, all cardiologists should be able to select the most appropriate imaging modality to solve each specific problem whilst aiming to avoid a standardized, undifferentiated sequential approach to the universe of available modalities without taking into account a preliminary detailed analysis and discussion in regard to which modality could be the most appropriate for each clinical situation and with which sequentiality, in a complementary step by step selective approach. Selected and appropriate indications should also help to overcome accessibility problems due to the uneven geographical distribution and availability of each technique for cardiology in different areas, often being in competition with other medical specialties for the access to each specific imaging modality.

This editorial would like to focus on the subject through the window of opportunity offered by the papers published last year in the *Journal of Clinical Medicine*.

## 2. Coronary Risk Prediction

Coronary artery disease (CAD) represents the most common cardiovascular disease, presenting with high morbidity and mortality. Historically, and following guidelines, patients with chest pain of suspected coronary origin have been assessed with functional tests capable of detecting the haemodynamic consequences of coronary obstructions and related prognosis through the depiction of electrocardiographic changes, myocardial perfusion defects or novel regional wall motion abnormalities under stress conditions [1]. Recent technical developments in stress echocardiography (SE), single-photon emission computed tomography (SPECT), positron emission tomography (PET) and cardiovascular magnetic resonance (CMR) have contributed to the increased diagnostic and prognostic performance of these techniques [1].More recently, cardiac computed tomography angiography (cCTA) has been developed as a noninvasive anatomical test allowing for a direct visualisation of coronary vessels and a detailed description of the atherosclerotic burden [1]. The accurate identification of individuals at high coronary risk could help to reduce acute coronary syndrome incidence and morbimortality. Coronary risk prediction in clinical practice was improved through the addition of coronary artery calcification and segment involvement scores, assessed using computed tomography, for the obtainment of cardiovascular risk factors [2]. Coronary artery calcification and segment involvement scores were associated with a five-year coronary event incidence, independent of cardiovascular risk factors [2]. In the research field, left ventricular myocardial and cavity Doppler velocity disturbances have been suggested to have the potential to become powerful predictors of significant coronary artery stenosis, as assessed using dobutamine stress Doppler echocardiography [3].

## 3. Myocardial and Pericardial Diseases

Myocardial and pericardial disorders encompass hypertrophic, dilated, arrhythmogenic, infiltratory, restrictive, inflammatory and other diseases of different aetiologies. Multimodality cardiac imaging techniques are extremely helpful in the definition of diagnosis, the assessment of prognosis and for monitoring the evolving natural history of these disorders through the planning of adequate follow-ups and treatments [4,5,6,7,8,9]. Transthoracic echocardiography should be the first-line imaging modality, due to its availability and repeatability. An important complementary role must be attributed to cardiovascular magnetic resonance (CMR), computed tomography and nuclear imaging, which are able to provide different types of accurate morphological and functional information, as well as extensive tissue characterization [4,5,6,7,8,9]. All this imaging information should be selectively incorporated into clinical decision making processes at the individual level, also taking into account the outstanding help clinical practice guidelines offer to clinicians [4,5,6,7,8,9]. Multimodality imaging is a comprehensive strategy for the investigation of left ventricular hypertrophy (LVH), providing morphologic, functional and often clinical information to clinicians [5]. Hypertrophic cardiomyopathy (HCM) is defined as an increased LV wall thickness, not only explainable through abnormal loading conditions. In the context of HCM, multimodality imaging conducted through different imaging techniques, such as echocardiography, cardiac magnetic resonance, cardiac computed tomography and cardiac nuclear imaging, provides essential information for reaching a diagnosis, sudden cardiac death stratification and management [5]. Furthermore, it is essential to uncover the specific cause of hypertrophic phenotypes, such as Fabry disease and cardiac amyloidosis, which can benefit from specific treatments [5]. In patients with inflammatory joint diseases and a mismatch between cardiac symptoms and routine noninvasive evaluations, CMR can uniquely identify a significant proportion of patients with myocardial inflammation [6]. The CMR-based assessment of the left atrial strain and left atrial strain rate could allow for a noninvasive diagnosis and the categorization of cardiac amyloidosis and its distinct differentiation from other hypertrophic phenotypes [7]. In the research field, scar imaging echocardiography through the ultrasound multipulse scheme has been shown to be able to detect subclinical myocardial involvement in systemic lupus erythematosus (SLE) in approximately one-fifth of SLE patients [8].

## 4. Heart Failure and Ventricular Function Assessment

Congestion is the main cause of hospitalization in patients with acute heart failure; however, its precise assessment with simple clinical evaluations remains elusive. The complementary assessment of clinical, natriuretic peptides and other laboratory parameters, together with echocardiographic and lung ultrasound scan information, can be implemented in clinical practice, allowing physicians to more precisely quantify pulmonary congestion in order to achieve a more complete temporal evaluation of the clinical picture, as well as to determine the prognostic power of each measurement or parameter [10].

Heart failure patients frequently develop brain deficits that lead to cognitive dysfunction. There is an important interaction between the brain and heart that becomes crucial for survival in patients with heart failure. An emerging experimental role of the combined brain/heart magnetic resonance imaging evaluation has the capability of diagnosing brain/heart lesions at an early stage and, potentially, facilitating their treatment [11]. Additionally, valuable complementary information regarding oedema, fibrosis and cardiac remodelling could improve heart failure risk stratification and treatment modification [11]. However, availability, familiarity with this modality and the cost should be taken under consideration before final conclusions can be drawn [11]. Abnormal cognitive dysfunction testing in HF patients can, however, be a strong motivating factor for applying combined brain/heart magnetic resonance imaging to identify early brain/heart lesions and modify risk stratification and treatments accordingly [11].

After a negative left ventricular remodelling, consequent to acute myocardial infarction, characterized by an increase in LV volumes and a depression in LVEF, operative treatment options can be considered to achieve a LV volume reduction and shape reconstruction. Conventional surgical LV reconstruction through a full median sternotomy has evolved towards the use of an experimental hybrid transcatheter and less invasive LV reconstruction [12]. A comprehensive understanding of all the technical considerations, individual procedural steps and adequate uses of multimodality imaging, both pre- and intraoperatively, is fundamental in order to perform a safe and effective hybrid LV reconstruction [12].

The assessment of the right ventricular morphology and function has gained increasing attention due to the prognostic importance of RV dilatation and dysfunction in patients with various cardiac and pulmonary diseases. A real-time evaluation of the right ventricular morphology and function through the use of three-dimensional echocardiography, combined with RV pressure-volume loops, could be useful in estimating RV-pulmonary arterial coupling, as previously experimentally assessed in an animal model [13].

The research assessment of left atrial function could be of clinical and prognostic relevance in restrictive cardiomyopathy and heart failure. A decrease in the left atrial reservoir strain, as assessed using CMR, has been shown to be independently associated with time to adverse events in patients with restrictive cardiomyopathy [14]. A left atrial overload measured with echocardiography is an important prognostic factor for heart failure readmission during the first year after enrolment in patients with HFpEF, but indices relating to a left atrial overload differed by sex [15].

Sarcopenia is a progressive and generalized disorder characterized by the accelerated loss of skeletal muscle mass, strength and functionality. It was found to be associated with an increase in morbidity and mortality in various conditions, such as cancer and orthopaedic and abdominal surgery [16]. The use of psoas cross-sectional measurements using manual CT segmentation before and after an endovascular aortic repair of an abdominal aortic aneurysm has been suggested for the monitoring of muscle depletion after surgery [16]. This interesting preliminary approach could also be of value and could possibly be further tested in aging frailty and end-stage heart failure and cachexia.

## 5. Valvular Heart Disease

Two-dimensional, three- dimensional and Doppler transthoracic and transoesophageal echocardiography are the first-line imaging modalities for the diagnostic and prognostic assessment of valvular heart disease (VHD), followed by cardiac magnetic resonance for tissue and flow characterization and the detection of myocardial and papillary muscle fibrosis [17,18,19,20,21,22]. Moreover, novel data have been reported on computed tomography and positron emission tomography utilizing 18F-fluorodeoxyglucose as a tool for providing evidence of early myocardial inflammation [17,18,19,20,21,22]. The introduction of other imaging modalities in the diagnosis of VHD could significantly improve our knowledge concerning cardiac mechanics; the tissue characterization of the burden imposed by the myocardium, calcium and inflammation on valves; and their impact on the severity, progression and prognosis of VHD, not only in symptomatic, but also in asymptomatic patients [17,18,19,20,21,22]. However, such a variety of novel parameters also brings uncertainty in regard to the clinical relevance of these indices, as well as the necessity for their validation in everyday practice. If we were to be able to positively elaborate on the diagnostic challenges and outline potential advantages of comprehensive multimodality cardiac imaging, we could better identify patients that could benefit from specific surgical or transcatheter valve interventions, as well as parameters that may be of help during follow-ups, for a more personalized and precision medicine [17,18,19,20,21,22].

The use of a transcatheter edge-to-edge repair for the treatment of mitral regurgitation has markedly increased in the last few years [19,23]. The rate of adverse events related to the procedure is low; however, some complications are potentially dangerous. Transoesophageal echocardiography has a key role in the guidance and monitoring of the intervention itself, allowing for the early diagnose of possible complications, including tamponade, thromboembolic events, single-leaflet device attachment, device embolization, vascular injury, etc. [23].

## 6. Infective Endocarditis

It is unclear whether the use of clinical prediction rules is sufficient to rule out infective endocarditis (IE) in patients with *Staphylococcus aureus* bacteraemia without an echocardiographic evaluation, either transthoracic and/or transoesophageal (TEE). The usefulness of PREDICT, POSITIVE, and VIRSTA scores to rule out infective endocarditis without echocardiography was assessed together with the secondary purpose of evaluating whether not performing an echocardiogram could be associated with higher mortality [24]. PREDICT and POSITIVE scores were not sufficient to rule out infective endocarditis without TEE [24]. In patients with a negative VIRSTA score, it was doubtful if infective endocarditis could be discarded with a negative TTE [24]. Not performing an echocardiogram was associated with worse outcomes, which might be related to the presence of occult IE [24]. Further studies are needed to assess the usefulness of clinical prediction rules for the avoidance of using echocardiographic evaluation in *Staphylococcus aureus* bacteraemia patients [24]. Besides echocardiography, a multimodality of imaging complementary approaches to IE, including cardiac magnetic resonance, computed tomography or positron emission tomography in combination with computed tomography (PET-CT), has gained interest in recent years [25,26,27], which could help reach a better understanding of the pathogenesis, clinical profile and outcomes in paediatric, adult and selective groups of IE [25,26,27]. Further research on multimodality imaging and the surgical treatment of IE is strongly needed to provide more comprehensive information for defining the most suitable treatment option, finding the optimal time for surgery and reducing morbidity and mortality.

## 7. Athlete’s Heart

Athlete’s heart refers to a wide spectrum of morphological and functional changes that occur in the heart and circulation of subjects practicing regularly physical activity, usually following a training program. When the morphofunctional changes associated with athlete’s heart are more relevant, they can overlap phenotypes mimicking structural cardiac diseases, including cardiomyopathies, valvular diseases, aortopathies, myocarditis and coronary artery anomalies. In these situations, a precise differential diagnosis is of paramount importance. This can be reached by applying an appropriate and selective approach to different imaging modalities, including rest and exercise stress echocardiography, speckle tracking echocardiography, cardiac magnetic resonance, computed tomography and nuclear scintigraphy [28,29]. In the presence of premature ventricular beats in athletes, cardiac magnetic resonance can be useful even when echocardiography is normal for the identification of nonischemic left ventricular scars and, potentially, arrhythmogenic substrates [30]. Imaging can be also used, together with other electrocardiographic techniques to differentiate athlete’s heart from pathological heart diseases, even though the overlap of different parameters is usually wide, sometimes complicating the distinction between health and disease in the individual case [29].

## 8. Congenital Heart Anomalies

Multimodality imaging is the basis for the diagnosis, follow-up and surgical management of congenital bicuspid aortic valve patients. The complementary role of transthoracic and transoesophageal echocardiography for valvular phenotyping and function, the measurement of thoracic aorta, exclusion of other aortic malformations, and assessment of complications such as infective endocarditis, aortic dilatation and dissection has been outlined [31,32]. In this setting, computed tomography and magnetic resonance could allow for a better precision of aorta size measurement through multiplanar reconstructions, a better definition of valve morphology and calcification, accuracy and reproducibility of ascending aorta size, an accurate assessment of coronary arteries, aortic dissection, aortic functional properties and blood flow patterns [31,32]. The integration of all multimodality information facilitates a comprehensive evaluation of morphologic and dynamic features for diagnosis, individualized stratification of associated risks and more precision therapy guidance of this cohort of patients [31,32].

The relationship between pulmonary regurgitation and ventriculoarterial interactions in patients with the postearly repair of the Tetralogy of Fallot was studied by using a cardiovascular magnetic resonance (CMR)-derived wave intensity analysis [33]. A correlation was found between aortic distensibility and the forward compression wave peak, suggesting unfavourable ventriculoarterial (VA) coupling in patients also presenting with stiffer ascending aortas [33]. Data suggest that VA coupling is affected by increased impedance [33].

The association of gestational age and echocardiographic markers as image modalities and levels of plasma N-terminal pro-B-type natriuretic peptides could help to predict the closure rate of a haemodynamically significant patent ductus arteriosus (PDA) [34]. Infants with spontaneous closure at seven days or less had significantly lower NTproBNP levels on day three compared with infants spontaneously closing later, infants treated either with ibuprofen only or surgery. Infants receiving PDA surgery later had significantly higher NTproBNP values on day three than other infants [34].

## 9. Left Atrium and Pulmonary Vein Isolation

Multiple cardiac imaging technique tools have been developed for the assessment of the left atrium. Although transthoracic echocardiography is the most widely employed procedure for the calculation of atrial size and function (due to its ease of use and wide availability), transoesophageal echocardiography, cardiac magnetic resonance, computed tomography and electroanatomical mapping can all be very useful for the management of cardiac rhythm disorders of atrial origin [35]. The multimodality imaging of the left atrium is mandatory for determining the most appropriate treatment for ailments of primarily atrial origin or involving the atria [35].

Endovascular pulmonary vein isolation (PVI) has become an important strategy for rhythm control in patients with symptomatic atrial fibrillation. Transseptal access is a critical step of this procedure, and can result in potentially life-threatening complications. TEE guidance may allow for safe transseptal access to the left atrium in patients undergoing PVI [36].
